# Concomitant occlusion of the deep dorsal vein and dorsal artery of the penis in a sickle cell anaemia patient

**DOI:** 10.1016/j.eucr.2026.103378

**Published:** 2026-02-16

**Authors:** Thomas Saliba, Arnaud Bourguignon, Guillaume Fahrni

**Affiliations:** a Erasme Hospital, Brussels, Belgium; b CHUV, Lausanne, Switzerland

**Keywords:** Mondor, Thrombosis, Artery, Sickle cell anaemia

## Abstract

Penile Mondor disease is a rare, often underrecognized condition caused by thrombosis of the superficial dorsal penile vein; arterial thrombosis is even rarer. We report a 30-year-old man with sickle cell anaemia who presented with 10 days of painful erections and dorsal penile induration. Doppler ultrasound demonstrated deep dorsal penile vein and left dorsal penile artery thrombosis. Sickle cell anaemia confers a hypercoagulable state predisposing to venous and arterial thrombosis. This represents the first reported case of combined deep dorsal penile vein and artery thrombosis, highlighting the value of Doppler ultrasound and need for clinical suspicion in patients with coagulopathies.

## Highlights

• Penile Mondor disease is rare and frequently underrecognized.
• First reported case of combined dorsal vein and artery thrombosis.
• Sickle cell anaemia may predispose to penile venous and arterial thrombosis.
• Doppler ultrasound is key for diagnosis and vascular assessment.

## 1 Introduction

The thrombosis of the superficial veins of the penis, also known as Mondor disease, was first described by Mondor in 1939, with Falco describing it in the context of generalized thrombosis in 1958 and Helm publishing a report of a case of isolated phlebitis the same year.^1^, ^2^, ^3^ Very few reported cases of this entity exist, with fewer than 50 reported as of 2010.^3^ Mondor disease is most commonly found in men aged between 20 and 70 years old.^4^

The condition, which can be either painful or painless, presents as cord-like induration on the dorsum or dorsolateral side of the penis.^5^ The pathogenesis is unknown, but it has been associated with trauma, mechanical stress, vigorous sexual intercourse, sexual abstinence, local infection, drugs, vacuum erection devices, pelvic tumour or coagulopathy as part of a SARS-COV-19 infection or sickle cell anaemia.^3^^,^^6^, ^7^, ^8^.

Occlusion of the dorsal penile arteries are rarer still, with few isolated case reports that report complications such as necrosis of the glans of the penis when both arteries are simultaneously occluded.^9^

We report the case of concomitant thrombosis of the dorsal vein and one of the two dorsal arteries of the penis in a patient suffering from sickle cell anaemia.

## 2 Case report

A 30-year-old man was urgently referred to the radiology department due to the appearance of a firm mass of the dorsal portion of the penis by his haematologist.

His medical history revealed that he suffered from sickle cell anaemia and had previously had a pulmonary embolism. The rest of his medical, surgical and family history did not reveal any relevant history.

The patient reported having experienced priapism and pain upon having erections which had been ongoing for the last 10 days. He also reported a palpable induration, which he identified as a vein, on the dorsal aspect of the penis. Any lateral movement of the patient's penis caused pain, which was accentuated when the patient was bending forward due to the mechanical constraints upon the hardened dorsal vein of the penis. Upon initial presentation of the symptoms, the doctor prescribed Cyproterone Acetate, to avoid the painful erections, with the hope that the issue would be self-limiting.

**Fig. 1:**
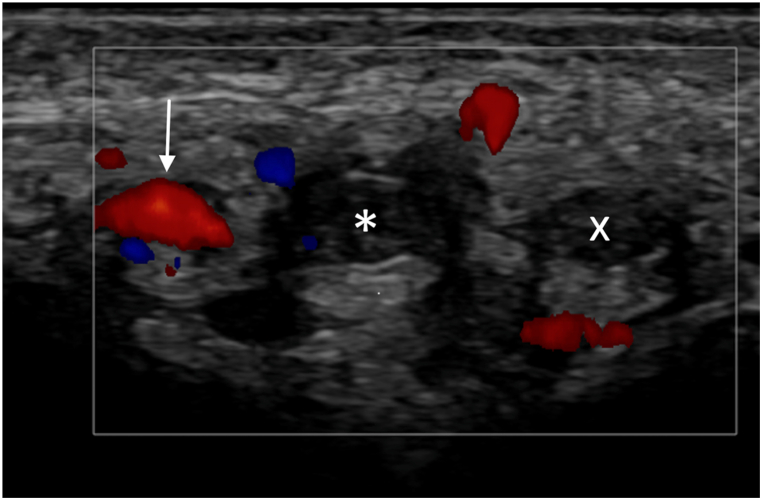
Doppler ultrasound image acquired using a 15MHz linear ultrasound probe showing hyperechoic blood clot within the dorsal penile vein (white star) and the left dorsal penile artery (white cross). There is a persistent doppler signal in the right dorsal penile artery (white arrow).

**Fig. 2:**
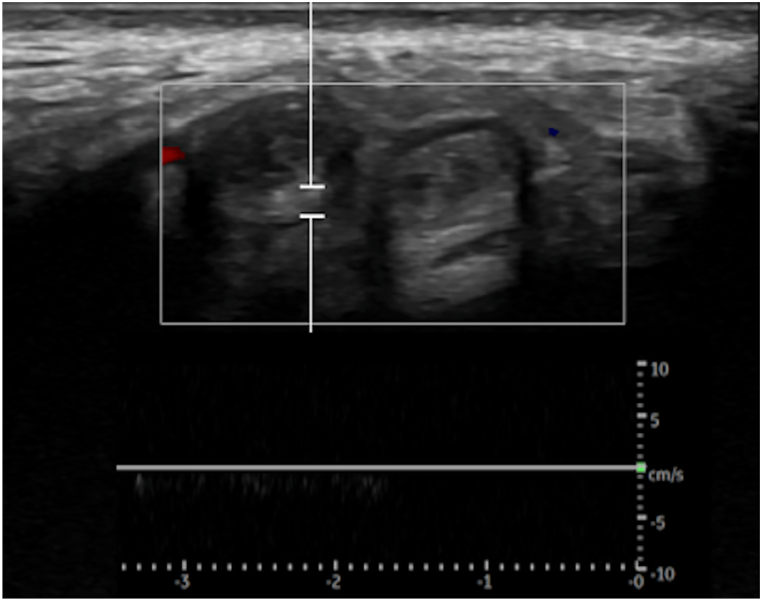
Pulse wave doppler ultrasound image showing hyperechoic blood clots within the dorsal penile vein and the left dorsal penile artery. A lack of flow within the dorsal penile vein is confirmed by the lack of signal.

**Fig. 3:**
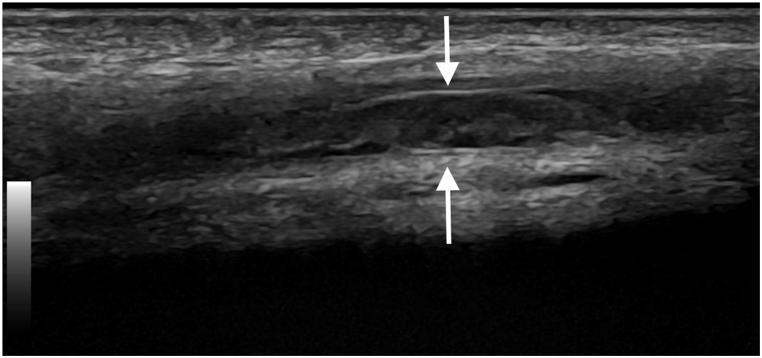
B-mode ultrasound image taken longitudinally along the dorsal vein of the penis, showing a hyperechoic thrombus distending the vein (arrows).

An ultrasound examination of the dorsal aspect of the penis was performed, revealing a thrombosis of the deep dorsal vein of the penis, extending 3cm to the base of the penis, confirming the suspected diagnosis. However, the patient also presented with an occlusion of the left dorsal penile artery at the same level (Fig. 1, Fig. 2, Fig. 3). The superficial dorsal vein did not have a thrombus. The central corporal arteries and the tissues of corpus cavernosum were unaffected.

The patient was prescribed apixaban, leading to the dissolution of the thrombosis and subsequent resolution of the pain two weeks after initial presentation. The patient continues to undergo monthly clinical follow-ups, with no recurrence having been reported.

## 3 Discussion

Thrombosis of the dorsal penile vein, also known as Mondor's disease, is very seldom reported in the literature, with fewer than 100 cases, though it is likely to have been under-reported.^8^ Due to the lack of reporting of this entity, it is poorly known to physicians, with one report. To our knowledge, only a single other case of this pathology in a patient with sickle cell anaemia has been reported.^8^

Sickle cell disease is a heritable haemoglobinopathy responsible for haemolytic anaemia, pain crises and organ damage in affected patients.^10^ When the red blood cells of patients with this disease are subjected to certain conditions, such as deoxygenation, they can undergo polymerization, which can promote thrombosis.^10^ In this case it is possible the hypercoagulable state caused by this condition contributed to the formation of a venous thrombosis.

Thrombosis of the dorsal penile artery is a very rare condition, with no cases of this spontaneously occurring available in the existing literature to our knowledge.

In this patient it is possible that, due to the hypercoagulability inherent to sickle cell anaemia, concomitant venous and arterial thrombosis occurred. Alternatively, it may also be possible that the vein was the first to have a thrombus, with the inflammation of the surrounding area causing an arterial thrombus to form.

Three stages of Mondor disease are described. The first is the induration phase, with the appearance of a cord-like hardening on the dorsal aspect of the penis.^4^ The second phase occurs one week after the initial presentation, with lessening of the pain and the appearance of irritative urinary symptoms.^4^ The final phase occurs around 9 weeks after onset and involves the breakdown of the thrombus and restoration of blood flow within the vein.^4^

Although the diagnosis mainly relies on a thorough medical history and physical examination, doppler ultrasound can be used to confirm the diagnosis by demonstrating a lack of blood flow and a thrombus within the dorsal penile vein.^11^ Furthermore, the affected vein may also present with inflammatory changes of the walls.^12^

The treatment of Mondor disease is supportive, involving sexual abstinence, warm compresses and non-steroidal anti-inflammatory medication and topical anticoagulants.^12^ If there is concern over a potential infection then topical antibiotics can be given.^12^ However, in cases of extreme pain, surgical thrombectomy can be performed, with one case report existing of long-term warfarin treatment to avoid recurrence in a patient with a coagulopathy.^13^

The differential diagnosis includes sclerosing lymphangitis and Lapeyronie's disease, which should both be considered in cases of painful fibrotic disease of the penis.^14^

## 4 Conclusion

We report the first case of concomitant thrombosis of the deep dorsal penile vein and a dorsal penile artery, occurring in a man with sickle cell anaemia. This case sheds light on the poorly known and likely underreported condition of Mondor disease, demonstrating the need to include it in the differential diagnosis of dorsal penile indurations, particularly in patients with sickle cell anaemia and other coagulopathies.

## CRediT authorship contribution statement

**Thomas Saliba:** Writing – review & editing, Writing – original draft, Visualization, Project administration, Methodology, Investigation, Formal analysis, Data curation, Conceptualization. **Arnaud Bourguignon:** Writing – review & editing, Writing – original draft, Validation, Supervision, Investigation, Formal analysis. **Guillaume Fahrni:** Writing – review & editing, Writing – original draft, Validation, Supervision, Project administration, Investigation, Formal analysis, Conceptualization.

## References

[1] Braun-Falco O. (1955). Clinical manifestations, histology and pathogenesis of the cordlike superficial phlebitis forms. Dermatol Wochenschr.

[2] Helm J.D., Hodge I.G. (1958). Thrombophlebitis of a dorsal vein of the penis: report of a case treated by Phenylbutazone (Butazolidin). J Urol.

[3] Nazir S.S., Khan M. (2010). Thrombosis of the dorsal vein of the penis (Mondor's disease): a case report and review of the literature. Indian J Urol.

[4] Ocampo M.A., Chavarriaga J., Fakih N., Silva J.M. (2019). Mondor disease - an underdiagnosed pathology: case report and review of literature. Clin Res Urol.

[5] Polat H., Cift A., Gulacti U., Benlioglu C. (2015). The damage of penile doppler ultrasonoghraphy in diagnosis of penile mondor's disease: a report of two cases. Urol Case Rep.

[6] Bagheri S.M., Tabrizi Z. (2021). Deep dorsal penile vein thrombosis in a patient with COVID‐19 infection: a rare complication and the first reported case. Clin Case Rep.

[7] Nachmann M.M., Jaffe J.S., Ginsberg P.C., Horrow M.M., Harkaway R.C. (2003). Sickle cell episode manifesting as superficial thrombophlebitis of the penis. J Am Osteopath Assoc.

[8] Ouattara A., Paré A.K., Kaboré A.F. (2019). Subcutaneous dorsal penile vein thrombosis or penile Mondor's disease: a case report and literature review. Case Rep Urol.

[9] Camacho J., Grand R., Blevins N., Sharma P., de Riese W., Cammack J. (2018). Occlusion of bilateral dorsal penile arteries resulting in glans necrosis in an obese male truck driver. Radiol Case Rep.

[10] Mangla A., Agarwal N., Maruvada S. (2025).

[11] Bressan M., Tessari M., Cosacco A.M., Zamboni P. (2021). Mondor's disease of the penis due to asymptomatic infective prostatitis provoking episodes of secondary sclerotizing lymphangitis. Veins and Lymphatics.

[12] Saraiva C.B., Campos N., Donato P. (2025). Penile Mondor's disease: an exceedingly rare diagnosis. Eurorad.

[13] Shen H.-L., Liu S.-P., Wang S.-M., Tsay W., Hsieh J.-T. (2007). Elevated plasma factor VIII coagulant activity presenting with thrombophlebitis of the deep dorsal vein of the penis. Int J Urol.

[14] Linden-Castro E., Pelayo-Nieto M., Ramirez-Galindo I., Espinosa-Perezgrovas D., Cornejo-Davila V., Rubio-Arellano E. (2018). Mondor disease: thrombosis of the dorsal vein of the penis. Urol Case Rep.

